# Retraction: Mitochondrial Dysfunction Promotes Breast Cancer Cell Migration and Invasion through HIF1α Accumulation via Increased Production of Reactive Oxygen Species

**DOI:** 10.1371/journal.pone.0205288

**Published:** 2018-10-02

**Authors:** The PLOS ONE Editors

In this article [1], concerns were raised about similarities between the following figure panels:

- Fig 2A, A clone + NAC cells and Fig 6D, A clone
- Fig 3A, A clone at 0 h and Fig 3A, B clone + NAC at 24 h
- Fig 2B, 4T1 cells and Fig 2B, C clone + NAC
- Fig 4C and 4D, H_2_O_2_ panels
- Fig 4D, control and Fig 6C, A clone
- Fig 4C, Mito-TEMPO and Fig 6D, A clone shRNA
- Fig 6C, A clone shRNA and Fig S2A, B clone
- Fig 5B, β-actin lanes 1, 2 (labelled “4T1 cells” and “C clone”, respectively) and Fig 6B β-actin lanes 1, 2 (labelled “A clone” and “A clone shRNA”, respectively)
- Fig 5B β-actin lane 5 (labelled “D clone + NAC”) and Fig 6B HIF-1α lane 1 (labelled “A clone”)

In addition, it was noted that in Fig 3B, the 24 h panels each have clusters of cells that appear highly similar to and in the same location as cell clusters in the 0 h panels, with additional cells present in the 24 h panel. There are also cell clusters similar in appearance and location in the 0 h, 24 h, and 48 h data for the C clone, and in the 24 h and 48 h data for C clone + NAC.

Vertical discontinuities suggestive of image splicing were also noted in Fig 5B between lanes 1 and 2 in the Hypoxic HIF-1α blot.

A Correction was published in 2014 in which the authors provided new panels for Fig 3A (B clone + NAC, 24 h), and for the Western blot and cell images in Fig 6B, C, D [2].

Wayne State University investigated this work and found evidence of data duplications, image adjustments, and data mislabeling in Figs 2, 4, 5, and 6. The corresponding author did not provide the original data and records to the investigating committee.

In light of these concerns, and in line with the institution’s recommendation, the *PLOS ONE* Editors retract this article.

QY and CC agreed with the retraction. JM, QZ, SC, BF, LM, FHZ, JX and ZW did not respond.
